# First Evidence of Genetic Intraspecific Variability and Occurrence of *Entamoeba gingivalis* in HIV(+)/AIDS

**DOI:** 10.1371/journal.pone.0082864

**Published:** 2013-12-20

**Authors:** Sibeli B. S. Cembranelli, Fernanda O. Souto, Kennio Ferreira-Paim, Túlio T. Richinho, Poliana L. Nunes, Gabriel A. N. Nascentes, Thatiana B. Ferreira, Dalmo Correia, Eliane Lages-Silva

**Affiliations:** Infectious and Parasitic Diseases Unit and Parasitology Unit, Federal University of Triangulo Mineiro, Uberaba, Minas Gerais, Brazil; Universidade Federal de Minas Gerais, Brazil

## Abstract

*Entamoeba gingivalis* is considered an oral commensal but demonstrates a pathogenic potential associated with periodontal disease in immunocompromised individuals. Therefore, this study evaluated the occurrence, opportunistic conditions, and intraspecific genetic variability of *E. gingivalis* in HIV(+)/AIDS patients. *Entamoeba gingivalis* was studied using fresh examination (FE), culture, and PCR from bacterial plaque samples collected from 82 HIV(+)/AIDS patients. Genetic characterization of the lower ribosomal subunit of region 18S (18S-SSU rRNA) was conducted in 9 positive samples using low-stringency single specific primer PCR (LSSP-PCR) and sequencing analysis. *Entamoeba gingivalis* was detected in 63.4% (52/82) of the samples. No association was detected between the presence of *E. gingivalis* and the CD4^+^ lymphocyte count (≤200 cells/mm^3^ (*p* = 0.912) or viral load (*p* = 0.429). The LSSP-PCR results helped group *E. gingivalis* populations into 2 polymorphic groups (68.3% similarity): group I, associated with 63.6% (7/11) of the samples, and group II, associated with 36.4% (4/11) of the samples, which shared 74% and 83.7% similarity and association with C and E isolates from HIV(−) individuals, respectively. Sequencing of 4 samples demonstrated 99% identity with the reference strain ATCC 30927 and also showed 2 divergent clusters, similar to those detected by LSSP-PCR. Opportunistic behavior of *E. gingivalis* was not detected, which may be related to the use of highly active antiretroviral therapy by all HIV(+)/AIDS patients. The high occurrence of *E. gingivalis* in these patients can be influenced by multifactorial components not directly related to the CD4^+^ lymphocyte counts, such as cholesterol and the oral microbiota host, which could mask the potential opportunistic ability of *E. gingivalis*. The identification of the 18S SSU-rRNA polymorphism by LSSP-PCR and sequencing analysis provides the first evidence of genetic variability in *E. gingivalis* isolated from HIV patients.

## Introduction

Oral manifestations are frequent in human immunodeficiency virus (HIV) patients and are primarily and easily diagnosed during the course of HIV infection. Oral manifestations are diagnosed and classified according to guidelines developed by the EC-Clearinghouse on Oral Problems Related to HIV Infection and the World Health Organization Collaborating Center on Oral Manifestations of the Immunodeficiency Virus. However, the oral parasite *Entamoeba gingivalis* is not included as an HIV-associated periodontal infection in the current guidelines [1].

*Entamoeba gingivalis* was originally isolated and described by Gros [2], but subsequent studies on this parasite are scarce, outdated, and controversial, mainly due to the difficulty in maintaining *E. gingivalis in vitro*. *Entamoeba gingivalis* exists as a trophozoite and is transmitted through oral contact. Its occurrence can vary according to age, presence of periodontal disease, and immunosuppression conditions [3], [4], [5].

*Entamoeba gingivalis* is considered a harmless commensal organism in humans and is commonly found in the calculus and bacterial plaques, crevicular fluid, and saliva [6], [7], [8], [9]. There are controversies concerning its pathogenicity because it has been detected in healthy individuals but has also been associated with periodontal disease [10].

Periodontal disease is a major complication of HIV infection [11] and occurs due to changes in cellular immunity and the production of metabolites that may influence the proliferation of non-periodontal pathogens in pockets in such individuals [12]. Additionally, a higher prevalence of opportunistic microorganisms has been detected in the subgingival microbiota of HIV(+)/AIDS patients, and other microorganisms, such as *E. gingivalis*, have the potential to turn into opportunistic pathogens [13], [14]. Although this behavior has not been observed with *E. gingivalis*, there is a suggested association between *E. gingivalis* and immunocompromised patients [5], [15], [16], [17]. Moreover, the pathogenic and opportunistic potential of *E. gingivalis* has been demonstrated experimentally by lesion development in immunosuppressed animals [18], [19], [20].

Little is known about the genetic [21], [22] and biological characteristics [18], [19], [20] of *E. gingivalis* as well as its role as a facilitator of oral lesions that contribute to the onset and progression of periodontal disease in HIV(+)/AIDS patients. However, studies conducted with *Entamoeba histolytica*, whose pathogenicity is associated with genotypic [21], [23] and phenotypic characteristics [24], may provide evidence that pathogenicity can occur with *E. gingivalis* populations.

The association of polymorphic populations of *E. gingivalis* with different levels of pathogenicity and or opportunistic behavior is still unknown. Therefore, this study evaluated the occurrence, possibility of opportunistic conditions, and intraspecific genetic variability of *E. gingivalis* in HIV(+)/AIDS patients.

## Methods

### Patients

The study included 82 HIV(+)/AIDS patients (51 male and 31 female; average age, 40.49±10.52 years) from Triangulo Mineiro region, Minas Gerais State, Brazil. The patients were treated at the Clinic of Infectious and Parasitic Diseases of the Hospital Clinics of the Federal University of Triangulo Mineiro – UFTM (Uberaba, Minas Gerais, Brazil), which is considered a regional reference center for AIDS. The patients were submitted to a full mouth examination including gingival aspect, the number of teeth, tooth mobility, presence of gingival bleeding, visible plaque, presence of visibly carious lesions, brushings number, flossing, mouthwashes and harmful habits and a periodontal probing (Hu-Friedy®). The patients were classified with gingivitis based on gingival aspects, biofilm presence, bleeding on probing and probing depth ≤3 mm and periodontitis with a probing depth ≥4 mm [25]. Oral sampling sites were randomly chosen depending on the number of teeth in the oral cavity. The exclusion criterion was edentulous patients with or without complete dentures. This study was conducted according to ethical principles for human research and Declaration of Helsinki and was approved by the Ethics Committee of the Federal University of Triangulo Mineiro under protocol number 1.377. All patients have provided and signed their written informed consent and answered a questionnaire to obtain socio-demographic, general and oral health conditions.

### Sample Collection and Processing

Bacterial plaque samples were collected by scraping along the gumline using a sterile toothpick and transferred to tubes containing 1 mL sterile saline (0.9% NaCl) and concentrated for 20 s at 7,840 × *g*. The pellet was resuspended in 350 µL sterile saline (0.9% NaCl) and then used for FE, culture, and molecular techniques.

### Parasitological Diagnosis by FE and Culture

The FE was conducted on microscope slides adding 5 µL in a coverslip and examined at 40× magnification for visualization of trophozoites. The culture was performed in Boeck-Drbohlav biphasic medium (BDM) [26] pH 6.7 modified in this study by buffering with 25 mM HEPES (Gibco). An inoculum of 100 µL were incubated at 35°C [19], [27], and subcultures were performed every 48 h. After the first 48 h of cultivation, a 0.6% rice starch solution containing penicillin (1.000 UI/mL), streptomycin (500 µg/mL) and nystatin (12 UI/mL) was added to the liquid phase of the medium.

### Specific Amplification of 18S-SSU rRNA of *E. gingivalis* by Conventional PCR

Two hundred microliters of *E. gingivalis* pellet was used in DNA extraction using phenol/chloroform technique [28]. The PCR reaction was performed in a final volume of 25 µL containing the following: 1× buffer (10 mM Tris-HCl pH 8.5; 50 mM KCl), 1.5 mM MgCl_2_, 0.2 mM of each dNTP, 5 pmol of each primers EGO-1 (5′-GAATAGGCGCATTTCGAACAGG-3′) and EGO-2 (5′-TCCCACTAGTAAGGTACTACTC-3′), 2.5 U *Taq* DNA polymerase (Platinum Invitrogen), and 3 µL of genomic DNA. The amplification program was performed as previously described [22].

The amplified products (1,400 bp) were visualized by electrophoresis on a 6% polyacrylamide gel stained with 0.2% silver nitrate.

### Genetic Variability of 18S-SSU rDNA from *E. gingivalis* by Low-stringency Single Specific Primer PCR (LSSP-PCR)

**Figure 1:**
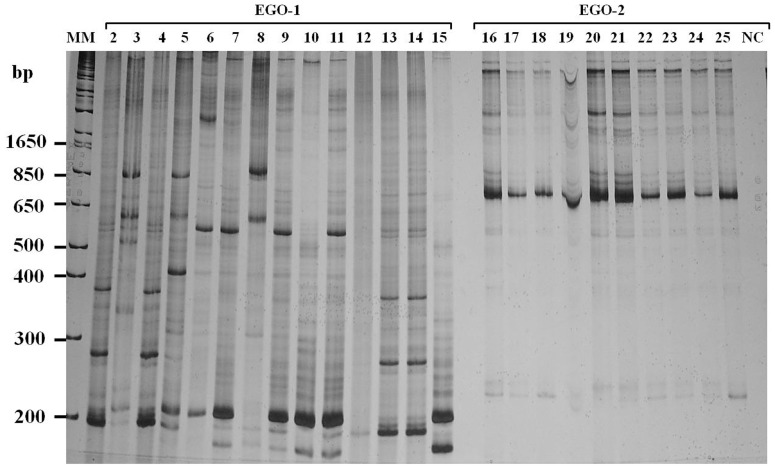
LSSP-PCR. Polyacrylamide gel (7.5%) stained with silver nitrate. Primers: A) EGO-1 and B) EGO-2. Lane 1: molecular marker 1 Kb (Invitrogen); lanes 2–25: HIV(+)/AIDS samples; lane 26: negative control.

LSSP-PCR was performed on samples from 9 HIV(+)/AIDS patients and 2 HIV(−), controls individuals. Approximately 15 ng of the *E. gingivalis* PCR product (1,400 bp) [29] was purified on a 1.5% agarose gel (1.0% agarose and 0.5% low-melting agarose), diluted 1∶10, and used as template DNA for LSSP-PCR [30]. The DNA samples were reamplified using a single specific primer as a driver corresponding to EGO-1, [22] which was previously selected to allow detection of genetic variability of the SSU region of the 18S rDNA of *E. gingivalis* (Figure 1). The 12- µL reaction mixture contained 1 × buffer (10 mM Tris-HCl pH 8.5; 50 mM KCl), 1.5 mM MgCl_2_, 0.2 mM of each dNTP, 1.0 U of *Taq* DNA polymerase (Cenbiot Ludwig), 45 pmol EGO-1 primer, and 3 µL template DNA. The amplification program was performed as previously described [30]. The LSSP-PCR products were separated by electrophoresis on a 7.5% polyacrylamide gel and stained with 0.2% silver nitrate. The electrophoresed bands were analyzed by visual scan photography of the gel. The proportion and construction of the phenograms were calculated using Gel Compar II software (Applied Maths NV).

### Sequencing of *E. gingivalis* 18S-SSU rDNA

The purified PCR products for 18S-SSU rDNA of *E. gingivalis* [22] were sequenced with forward and reverse primers directed to EGO-1 and EGO-2, using BigDye Mix® (BigDye Terminator Kit, version 3.1, Applied Biosystems) in a DNA sequencer (ABI PRISM® 3130 XL Genetic Analyzer, Applied Biosystems) according to the manufacturer’s protocol.

The sequences were edited with Sequence Scanner V. 1.0 software (Applied Biosystems) using the consensus sequences originating from the forward and reverse primers. The neighbor-joining method [31] for calculating evolutionary distance was performed according to Kimura’s method [32]. The reliability of each node of the phylogenetic tree was estimated by the bootstrap method using 1,000 replicates. Phylogenetic analysis was performed with MEGA 5.0 software [33] using a comparative standard for *E. gingivalis,* nucleotide sequences of the reference strain ATCC 30927 (American Type Culture Collection), and sequences corresponding to several different amoeba species that were obtained from GenBank and identified by their accession number. The nucleotide sequences here discussed have been deposited in the GenBank (accession number KF250433 to KF250436).

### Quantification of CD4^+^ Lymphocytes and Viral Load of HIV(+)/AIDS Patients

Whole blood was subjected to flow cytometry (BD FACS CALIBUR, Becton Dickinson) with BD MultiTEST® CD3-FITC/CD8-PE/CD45-PerCP/CD4-APC (Becton Dickinson), analyzed with MultiSet software, and classified in >200 and <200 cells/mm^3^ of blood.

HIV-1 RNA in plasma was directly quantified using VERSANT® HIV-1 RNA 3.0 - bDNA (Siemens). Viral RNA amplification was classified in terms of 5 scales of mRNA copies/mm^3^: <50; 51–5,000; 5,001–30,000; 30,001–100,000; and >100,000. The low and high limits of quantification correspond to 50 and 500,000 copies/mL of RNA/HIV-1, respectively.

### Statistical Analysis

The association between FE and PCR positivity for *E. gingivalis* and each of the categorical variables studied was analyzed using the chi-square test, and the agreement between both was evaluated by calculating the Kappa coefficient.

## Results

### Occurrence of *E. gingivalis* in HIV(+)/AIDS Patients

The occurrence rate of *E. gingivalis* determined by direct FE and/or PCR was 63.4% (52/82). The occurrence rate significantly differed between the PCR and FE methods, corresponding to 56.1% (46/82) and 36.6% (30/82), respectively (*p = *0.013; OR = 2.21 [1.18–4.14]). The kappa concordance obtained by PCR and FE was 65.9% (54/82) (*k* = .34 [0.14 to 0.54], 95% CI) with the positive and negative concordance being 29.3% (24/82) and 36.6% (30/82), respectively.

*Entamoeba gingivalis* was detected in 63.8% (37/58) and 62.5% (15/24) of patients with CD4^+^ lymphocyte counts >200 cells/mm^3^ and ≤200 cells/mm^3^, respectively (*p = *0.912). The parasite was detected in 56.1% (23/41), 82.4% (14/17), 60% (6/10), 50% (1/2), and 66.7% (8/12) of patients who had <50, 51–5,000, 5,001–30,000, 30,001–100,000 and >100,000 copies of the virus, respectively (*p = *0.429).

### Entamoeba Gingivalis Cultivation

Of the 82 samples collected, only 37 remained in cultivation. The BDM buffered medium allowed *E. gingivalis* growth in 29.7% (11/37) of the samples in culture for an average of 13.27±5.85 d. The multiplication ability of the isolates differed for each sample.

### Genetic Variability of *E. gingivalis* 18S-SSU rRNA

**Figure 2:**
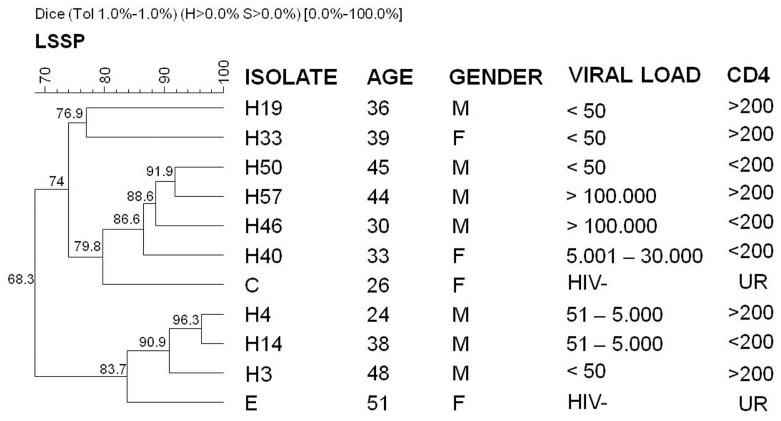
Analysis of the genetic variability of the small ribosomal subunit in the 18S rRNA of *E. gingivalis* in clinical samples from HIV(+)/AIDS patients, detected by LSSP-PCR. M = male; F = female; CD4 =  CD4^+^ cell count; HIV− = HIV negative; UR = unrealized.

The phenogram representing the genetic profiles of LSSP-PCR demonstrated that the samples of *E. gingivalis* were grouped into 2 divergent clusters, which shared 68.3% band similarity. Cluster I was associated with the C isolate from HIV(−) individuals and grouped 63.6% (7/11) of the samples, which shared 74% similarity. Cluster II was associated with the E isolate and grouped 36.4% (4/11) of the samples with 83.7% similarity. Joint analysis of the samples revealed a higher similarity between pairs H4 and H14 (96.3%) and H50 and H57 (91.9%) isolates (Figure 2). Analysis of isolates exclusively from HIV(+)/AIDS patients showed 66.1% band similarity (data not shown), and the phylogenetic tree maintained the same distribution demonstrated in figure 2. Comparative analysis of the distribution of the *E. gingivalis* samples on tree clusters with epidemiological and clinical characteristics of patients did not demonstrate any association related to age, gender, geographical origin (data not shown), CD4^+^ lymphocyte counts, and viral load.

### Sequencing of *E. gingivalis* 18S-SSU rDNA

During the process of sequencing the 18S-SSU rDNA, a 604-bp product was generated. A BLAST search confirmed the identity of *E. gingivalis* in the 4 isolates analyzed, isolates C and E obtained from immunocompetent individuals, and other isolates from the HIV(+)/AIDS patients. The BLAST search demonstrated that all isolates shared 99% maximum identity and 100% query coverage with ATCC 30927.

**Figure 3:**
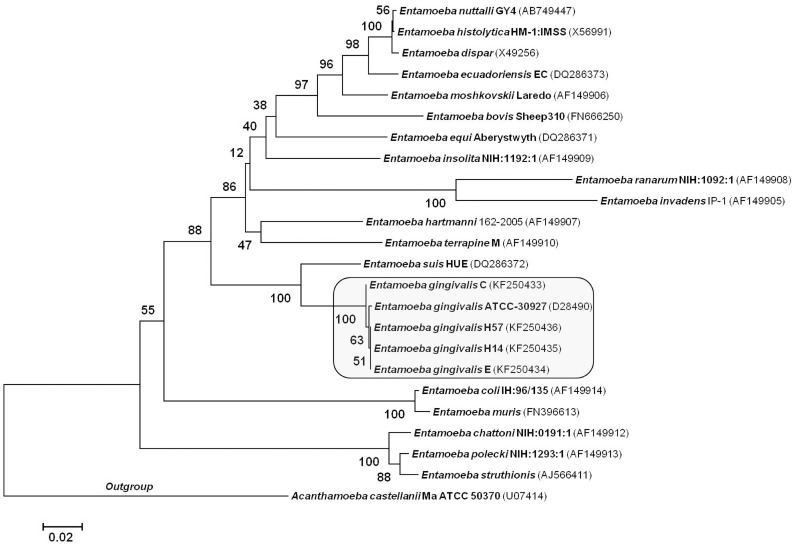
Phylogenetic relationship of *Entamoeba* spp. from HIV infected patients. Neighbor-joining method was performed in 24 taxa at the SSU rRNA locus. The optimal tree with the sum of branch length = 1.32 is shown and the bootstrap values were added to phylogenetic branches.

**Figure 4:**
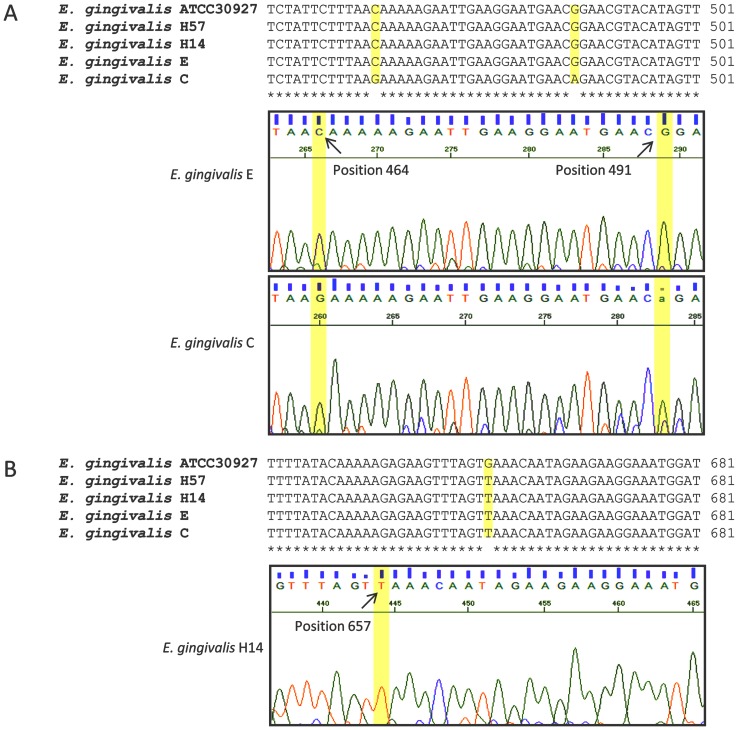
Single nucleotide polymorphisms in *E. gingivalis* isolates.

According to the phenogram, the samples were divided into 2 divergent clusters: 1) cluster I, which associated with isolate C and 2) cluster II, which associated with isolates E, H14, H57, and ATCC 30927 (Figure 3). The electropherogram analysis demonstrated that the C isolate from *E. gingivalis* differed in 3 positions from the ATCC 30927 strain and confirmed the distribution of this isolate in a different branch of the tree. The remaining isolates presented a single nucleotide polymorphism (SNP) at nucleotide 657 of the 18S gene when compared with ATCC 30927 (Figure 4). Comparison of the *E. gingivalis* sequences with those of amoeba species showed genetic identity of 83.1% with the *Entamoeba suis* Hue strain.

This distribution was similar to that observed by LSSP-PCR in which the C and E isolates were grouped into different clusters. However, the H57 isolate fragment analyzed by sequencing (604 bp) was different from that observed by LSSP-PCR (1,400 bp).

## Discussion

Although *E. gingivalis* pathogenesis remains unclear, HIV-affected individuals show accelerated development of chronic periodontitis, which could be the result of the patient’s oral microbiota and/or degree of immunodeficiency [12]. *Entamoeba gingivalis* is considered a commensal parasite of the mouth and its presence has been associated with compromised periodontal pockets, but it is not detected in healthy regions of the oral cavity of the same individual [10]. However, *E. gingivalis* is not included as a cause of oral lesions in HIV/AIDS by the guidelines of the EC-Clearinghouse on Oral Problems Related to HIV Infection and the World Health Organization Collaborating Center on Oral Manifestations of the Immunodeficiency Virus (1993) [1].

Data relating to the co-infection of HIV/AIDS and *E. gingivalis* are scarce. The findings of *E. gingivalis* (63.4%) in HIV(+)/AIDS patients with periodontal disease presented here corroborate with the higher occurrence of *E. gingivalis* (22%) in these individuals when compared with only 7% in HIV(−) individuals [5]. Of the HIV(+)/AIDS patients in our casuistry, 100% presented with gingivitis and/or periodontitis by the adopted criteria, which reinforces the strong association of *E. gingivalis* with periodontal disease as detected in 77% of HIV(+)/AIDS subjects with periodontitis [5].

Low CD4^+^ lymphocyte counts are considered the main risk factor associated with the development of oral lesions in immunosuppressed individuals [34], and periodontal inflammation seems to be more severe in these individuals [35]. Published data suggest possible opportunistic behavior of *E. gingivalis* because the high parasite frequency and advanced periodontitis [18] have been correlated with low CD4^+^ lymphocyte counts [5] and with immunosuppression [16], [17]. Our results did not establish an opportunistic behavior of *E. gingivalis* in HIV(+)/AIDS co-infected patients because parasite occurrence was not associated with a low immune response (CD4^+^ lymphocyte count ≤200 cells/mm^3^). This incongruence may be related to the use of highly active antiretroviral therapy (HAART), which induces an increase in immune responses and reduction in the severity, course, and prevalence (47–85% to 32–46%) of periodontal lesions [36], [37].

Moreover, the high occurrence of *E. gingivalis* in HIV(+)/AIDS patients can be influenced by multifactorial components not directly related to the CD4^+^ lymphocyte counts, such as cholesterol and the oral microbiota host, which could mask the potential opportunistic ability of *E. gingivalis*. Hypercholesterolemia has emerged as a comorbidity after HAART [38] and could occur 8 weeks after therapy initiation [39]. Although dyslipidemia was not evaluated in our patients, all of them had received HAART for at least 6 months. Additionally, cholesterol is essential for *in vitro* growth of *Entamoeba* species [40] and is required to stimulate and maintain the virulence [41], pathogenicity [42], and phagocytic ability of *E. histolytica* [43] and may have contributed to the high *E. gingivalis* occurrence in this study.

Chronic periodontitis in HIV(+) patients may be associated with specific microbial interactions detected exclusively in these patients [44] and could facilitate *E. gingivalis* infection because an intimate relationship between bacteria, such as *Actinomyces,* and a protozoan presence has been demonstrated [15], [45].

The oral cavity has been rarely reported as a site of HIV transmission [46], [47]. However, oral lesions and periodontal disease could promote the shedding of HIV-infected blood into the oral cavity [48] increasing the possibility for HIV transmission. Additionally, phagocytic protozoa could favor the spread of HIV, as suggested for *Trichomonas vaginalis*, by ingesting cells infected with the HIV virus that then remain viable inside them [49], [50], [51]. In this context, the ability of *E. gingivalis* to phagocyte leukocytes could play an epidemiological role in the oral transmission of the HIV virus and must be investigated.

In this study, the use of conventional PCR for the diagnosis of *E. gingivalis* in clinical samples demonstrated greater sensitivity than FE (*p = *0.13), which agrees with previous data [22], [52]. In addition, PCR results of this study also suggest a higher parasite load in HIV(+)/AIDS patients (56.1%) when compared with HIV(−) individuals who showed a lower occurrence (27%) rate using the same technique [10]. However, the parasite load detected was more similar to that of HIV(−) individuals (69%) when more sensitive methodology (real-time PCR) was employed [10]. Despite the absence of PCR inhibitors in our DNA samples, PCR failed to detect 7.3% (6/82) of the FE positive samples. Considering that the primer sequence specificity is based exclusively on the reference strain ATCC 30927 [22], the evidence of genetic variability of *E. gingivalis* found in this study is important for evaluating the ability of the primers used to detect unique and mixed parasite infections and other different populations of *E. gingivalis*. In addition, we cannot exclude the remote presence of other amoebae species in the oral cavity [13], [53].

The *E. gingivalis* 18S-SSU rDNA polymorphisms were detected for the first time among HIV(+)/AIDS clinical samples by the use of LSSP-PCR. These samples were distributed in 2 divergent clusters (68.3% similarity), and each one was associated with 1 of the 2 strains isolated from the HIV(−) individuals. This distribution was not associated with the geographic origin, gender, viral load, or CD4^+^ lymphocyte counts of the individuals. Nevertheless, this finding corroborates the only data in the literature in which 4 strains of *E. gingivalis* were grouped into 2 ribodemes [54].

The sequencing of 18S-SSU rDNA from 4 samples of *E. gingivalis* samples showed that they were genetically correlated with the reference strain ATCC 30927 and showed 2 divergent clusters similar to that detected by LSSP-PCR. The tree topology, mainly of both HIV(−) isolates, was confirmed by polymorphisms in 3 positions on the C isolate and a single nucleotide polymorphism (SNP) at nucleotide 657 of the 18S gene on the other isolates. The different grouping of the H57 isolate on the tree generated by LSSP-PCR and sequencing analysis may be associated with evaluation of few sequences and weak bootstrap analysis.

Another important contribution of this study, which resulted in a novel strategy for *in vitro E. gingivalis* maintenance [55], was buffering the liquid phase of the BDM medium to maintain the optimum pH (6.7) for *E. gingivalis* proliferation and maintenance in xenic cultivation [56].

The 18S-SSU rDNA polymorphisms detected among the *E. gingivalis* populations may open new perspectives on studying *E. gingivalis*. Its genetic variability may be related with biological properties or act as genetic determinants of pathogenesis [18], [20]. Although opportunistic behavior of *E. gingivalis* was not observed, its high frequency in HIV(+)/AIDS patients may facilitate the development of periodontitis and could be included in the classification of HIV-related oral lesions. Moreover, *E. gingivalis* participation in HIV infection persistence and the possibility of oral transmission should be investigated.
